# Contrasting the primings between English and Chinese: To advance Hoey's theory of lexical priming from the perspective of culture psychology

**DOI:** 10.3389/fpsyg.2022.893120

**Published:** 2022-08-22

**Authors:** Luojia Wang

**Affiliations:** Department of Software, Dalian University of Foreign Languages, Dalian, China

**Keywords:** lexical priming theory, English-Chinese contrast, culture psychology, collocation, semantic association, colligation

## Abstract

The present study demonstrated that Hoey's promising and serviceable theory could serve as one that can describe and explain similarities and differences exhibited between English and Chinese. Three fundamental concepts as defined in the theory (collocation, colligation, and semantic association) were considered. The combinatory profile showed that: (1) different word choices were primed to constitute to a shared semantic association (2) no unique word combination containing the nodes was found, but high or low frequency use of particular clusters and collocates appearing at both sides to the node occurred; (3) it is claimed that the interpretation and explanation of priming similarities and differences between English and Chinese need to be extended to culture psychology.

## Introduction

Since the late 1990's, Michael Hoey has been exploring the concept of colligation. Stemming from his exploration, the theory of lexical priming (LPT) was developed. This theory proposed a radical new theory of the lexicon, which amounts to completely new theory of language based on how words are used in real world. Hoey showed in his theory that the phenomenon of “collocation” offers a clue to the way language is really organized. The term “collocation” is normally treated as the fundamental concept of corpus linguistics. Among the few corpus linguists who have attempted to combine psychology and corpus data together, Hoey was the first one who asked why collocation—the starting point of corpus linguistic research came into being. Per Hoey, collocation is a key factor in naturalness. It does not refer to the regular co-occurrence of words in close proximity to each other, but are words regularly associated in the mind. This psychological association contributes a text's cohesion (Halliday and Hasan, 1976).

The pervasiveness of collocation requires explanation. It must be a psychological concept and the most appropriate psychological concept is “priming” (Hoey, 2005). The term of priming was first used by Quillian in his series of papers published between 1961 and 1969. In these papers, priming was used to describe the process of what Quillian and other researchers named “retrieval from the semantic memory.” It was Neely (1976) who combined “priming” and “lexical” together. Prominent research by Scarborough et al. (1977, p. 14) mirrored and reinforced Neely's experiment in many respects, in that it showed that a prior presentation of a “stimulus word” (priming word) could affect the recognition speed of the later presentation (target word). Both Neely and Scarborough et al. seemed to indicate that a single exposure to a related word (or non-word) would be stored in one's head and, once the related trigger was given, a word belonging to the same semantic field would be retrieved from memory. The focus of this psycholinguistic discussion was the relationship between the prime and the target, “rather than the word *per se*” (Hoey, 2005, p. 8). Hoey noted priming in his theory, calling it “a property of word and what is primed to occur is seen as shedding light upon the priming item rather than the other way round” (Hoey, 2005). Halliday and Hasan referred to the process of collecting priming through encounters as forming a mental lexicon, but did not provide the underlying reason for why this phenomenon exists. Hoey (2005, p. 9) complemented Halliday and Hasan and explained some characteristics of priming in his theory:

Priming need[s] not be a permanent feature of the word or word sequence; in principle, indeed, it never is. Every time we use a word, and every time we encounter it anew, the experience either reinforces the priming by confirming an existing association between the word and its co-texts and contexts, or it weakens the priming, if the encounter introduces the word in an unfamiliar context or co-text or if we have chosen in our own use of it to override its current priming.

The definition of priming was quoted at length to demonstrate that Hoey's use of priming is distinct from that of some psycholinguists (e.g., Neely, 1976; Scarborough et al., 1977). Priming, in Hoey's theory, stresses the word itself (*per se*). There is no priming word to trigger a target word; priming exists at the moment an individual decides to use a word. While the attention in some of the psycholinguistic work is on the recognition effects of a particular prime on a particular target, LPT is more interested in the way the target may accumulate such primes.

Priming builds on insights gained from corpus linguistics, and applies the notion of psycholinguistics thereto. Psychology is invisible and works on a far subtler level than the reoccurrences of word patterns. However, by making an analogy between mental concordance and computer concordance, priming can be studied by examining corpus evidence to indicate “the kinds of data a language user might encounter in the course of being primed” (Hoey, 2005, p. 14). Corpus cannot unveil how the head neurons work in the process of individual primings but indicates which primings are shared by large numbers of speakers in a language community. Indeed, psycholinguistics has shown corpus-based studies can provide results almost identical (<95%) to results obtained from psycholinguistic experiments (Gries, 2005).

Priming is not conscious choices of language users. Yet it can be consciously strengthened, weakened, expanded or reassigned. As Pace-Sigge and Patternson (2017, p. xiv) summarized that “primings are prevalent but they are neither fixed or prescriptive: they reflect one's exposure and language use.” The idea of Hoey's lexical priming has been in existence for almost 20 years. It has provoked many researchers to expand and advance this theory in various dimensions (details to be proved in Section Literature review). The researchers are more likely to approach this psychological subliminal effect as a possible explanation for corpus-based studies. Priming is contextualized. The previous researchers seemed to restricted their studies within textual contexts. Since LPT has been always seeking to explain various linguistic phenomena from a psychological perspective, the context where primings depend on should be extended to psychological dimension. This paper is aimed at filling this theoretical gap by bringing the concept of culture in psychology into discussion. A cross-linguistic study was undertaken to uncover cross-culture variations in psychological processes. As a consequence of radical economic growth, culture has emerged as a critical concept. It has become a “de jour concept of the contemporary discourse around the world” (Kashima, 2015, p. 1). It is regaining its place in psychology. The primary objective of psychology is to “describe, explain, predict, and understand human mind and behavior” (Kashima, 2015, p. 2). Applying culture psychology to complement LPT from a cross-linguistic perspective can first extend the theory's ability of accounting for cross-linguistic similarities and differences; and, second, expand the concept of context, which defines one's priming.

## Literature review

It has been more than 20 years since the 2005 publication of Hoey's lexical priming theory (LPT), since when various applications and expansions have been made to advance this theory in many dimensions. Expansions and advances have been provided either by applying the theory to a new linguistic field (e.g., spoken English, see Pace-Sigge, 2013, 2017) or to account for a linguistic phenomenon (e.g., metaphor, see Patterson, 2016). A review of the literature applying LPT to languages other than English (e.g. Finnish, see Jantunen and Brunni, 2013; Jantunen, 2017; and Turks and Ottomans, see Baker et al., 2017) implies that examining a language genetically different from English could generate new insights.

Although LPT has been applied and expanded by exploring English and other languages, it has not been fully explored and tested with Chinese, with Hoey and Shao (2015) being among the few researchers to address this issue. Through a preliminary study of several notions defined in LPT—collocation, semantic association, and pragmatic association—Hoey and Shao convincingly showed the possibility that the characteristic features of Chinese could be demonstrated with corpus and accounted for with psycholinguistic theory, such as LPT. However, they were not able to extend their study to either colligation or the integration between colligation and other conceptual notions. This article aims to fill this gap by examining the given lexical items at three contextual levels—collocation, colligation, and semantic associations. The focus of this study is attempting to find evidence of this dependency relation in Chinese and then contrast the given nodes based on a combinatory profile, which have not been done in the literature of contrastive linguistics. Based on the results of the contrast, the present study also explored an aspect not yet tackled in neither psycholinguistics, corpus linguistics or contrastive studies, i.e., whether LPT could be adopted to account for the priming features between English and Chinese; and whether their characteristic features of priming can be accounted for by taking culture psychology into consideration. The specific research questions in focus are:

1. What are the differences and similarities of collocational, semantic associational, and colligational primings between English and Chinese?
2. To what extent can culture psychology approach to the description of the priming features demonstrated cross-linguistically?

## Research methodology

### The research nodes

The nodes of *world* and 世界 *shi4jie4* were chosen. The reasons are 3-fold. First, *world* and 世界 *shi4jie4* occur with a similar frequency in the two languages, with *world* occurring in the British National Corpus 520.94 times per million running words and 世界 *shi4jie4* occurring 512.45 times per million running words in zhTenTen11. The frequent use of the nodes in the two corpora implies numerous concordance lines will be found in various cases, which could offer ample data for its potential encounterable instances. Second, the body senses of *world* and 世界 *shi4jie4* are shared by speakers of the two languages. They use the nodes to describe not only the material or profane sphere but also the celestial, spiritual, transcendent, or sacred spheres. The similar understanding of the external and spiritual world inherited by the two groups of speakers may demonstrate, to a large extent, similar senses embedded within *world* and 世界 *shi4jie4*. The nodes of *world* and 世界 *shi4jie4* are likely to be culture-independent items whose semantic associations and prosodies are unlikely to be presumably identified for culture influence. Third, the nodes to be examined do not have multiple translational alternatives. The node pair of *world* and 世界 *shi4jie4* is highly equivalent to each other in terms of both literal and figurative meaning.

One of the research aims for many contrast linguists is to disclose some of the options people employ in the ways they conceptualize the world. They studied situations that are quasi-universal where different language speakers are exposed to. In the present study, the word “world” itself was chosen and studied from a cross-linguistic perspective. Results obtained in this respect will be more potential in the respect of underlying similarities and dissimilarities of conceptualization between English and Chinese.

### The corpora

Two kinds of corpora were used in the study. The first was reference corpora, including the British National Corpus (BNC) and the zhTenTen11 corpus, which were adopted to provide quantitative data. The BNC, created by Oxford University Press and completed in 1994, contains a 100-million-word collection of written and spoken samples drawn from a wide range of genres (e.g., fiction, magazines, newspapers, academia). The zhTenTen11 is a simplified modern Chinese corpus containing almost 2.6 million documents with more than 1.7 billion words in over 72 million sentences. The two reference corpora are freely available on the Sketch Engine website (https://auth.sketchengine.eu/).

The second kind was comparable corpora, including the Freiburg-LOB corpus of British English (henceforth FLOB) and the Lancaster Corpus of Mandarin Chinese (henceforth LCMC), which were applied to provide qualitative data. Having equivalent numbers of tokens and a similar sampling method, FLOB and LCMC were initially established for the cross-linguistic study of English and Chinese. English-Chinese cross-linguists have widely used the two corpora to examine numerous linguistic features (e.g., Xiao and McEnery, 2006, 2010).

A representative but manageable sample of instances was retrieved from the comparable corpora to investigate the potential colligations of *world* and 世界 *shi4jie4*. The instances were first coded by grammatical function (Subject, Object, Complement, or Adjunct) and then by their position in a nominal group (noun head, part of premodification, or part of postmodification).

### Research tools

In this study, two corpora analyzing tools were used: One was AntConc (3.5.9), freely downloadable software whose functions are all workable for Chinese. The other analytic tool was Sketch Engine, an ultimate corpus tool to create and search text corpora in many languages. In addition to its large capacity for corpora of different languages, Sketch Engines' new but a very reasonable statistic association score (LogDice) gives very good results for collocation candidates in a large corpus, focusing on the co-selecting strength of the node word and its collocates. LogDice is arguably “more reliable since it will not be biased by either too-high or too-low frequency of the items in the query” (Rychlý, 2008 cited in Hoey and Shao, 2015, p. 23).

### The research method

Both quantitative and qualitative methods were applied in this study. The former included the large concordance lines and statistics provided and calculated by Sketch Engine, and, the latter, the sampling instances retrieved from the comparable corpora. As mentioned in Section The corpora, each instance was coded with different grammatical categories, and then their frequencies and probabilities were calculated and compared. The research procedure is detailed below.

First, the general collocational behavior of *world* and 世界 *shi4jie4* was examined and compared. I explored and compared the pair's collocation and semantic associations, and then studied the clusters containing the nodes. Their collocates and semantic association actualized thereby were also examined. At last, I investigated the node pair's general colligational behavior; i.e., their preferences/avoidances^1^ for particular grammatical functions and positions.

## Findings and discussions

### The contrast of overall collocational and semantic associational behavior between *world* and 世界 *shi4jie4*

As has been discussed previously, Hoey's LPT intended to pick up the fact that collocation is a psycholinguistic phenomenon. Collocation, in his theory, is defined as “...a psychological association between words (rather than lemmas) up to four words apart and is evidenced by their occurrence together in corpora more often than is explicable in terms of random distribution” (Hoey, 2005, p. 5). Assuming that lexical priming shall not only operate regarding collocations and that a regular kind of lexical priming shall be considered, Hoey used “semantic association” to talk about the psychological preference on the part of the language user. It is defined as “...a word or word sequence is associated in the mind of a language user with a semantic set or class, some members of which are also collocates for that user” (Hoey, 2005, p. 24). According to Hoey, semantic association is a necessary generalization and could account for co-occurrences that cannot be explained in terms of collocation.

The present research began by retrieving a concordance of *world* with a span of four words to the left and right sides. A total of 58,496 instances were found in the BNC corpus, with 520.94 occurrences per million running words. Analyzing these 58,496 instances in Sketch Engine, I found a greater consistency of patterning and sets of variables to the left of the collocation than to its right. Since collocates at the R3/R4 position (the third/fourth position to the right of the node word) and L4 (the fourth position to the left of the node word) of *world* were too sparse to collect, they were not distributed in this article.

The collocates of the node word *world* were displayed in descending order of their LogDice scores; due to page limits, not all collocates were displayed.

At the L1 position, we can broadly group a set of words, including *Third, Second, First, third* and *second*, into a semantic association of RANK and the words *Western* and *Arab* into a semantic association of AREAS. At the L2 position, the collocates were dominated by prepositional words (which is also colligational behavior), including *around, throughout, outside, over, in, of*, *round, across*, and *into*, which showed that *world* was likely to appear in the form of frequent phrases. Although better categorized as grammatical words, these collocates could also be grouped into a semantic association of POSITIONING IN LOCATION (e.g., *around, outside, in*). The prepositions of *during* and *after*—not shown in the table, but still a frequent collocate at the L2 position of *world*, with a LogDice score of 5.074—seemed to be associated with a semantic meaning of POSITIONING IN TIME. At the L3 position, the collocates were accumulated to form three kinds of semantic meaning. The first one was that of POSITIONING IN LOCATION, represented by members of *parts, anywhere, Western*. The second one is that of RANK, represented by ordinal words of *second, third*, etc. The third one is that of SUPERLATIVE, made up of words, such as *best, largest*, etc. Then I continued the examination on 世界 *shi4jie4*. There were 1,079,563 instances of 世界 *shi4jie4* in the zhTenTen11 corpus. The data demonstrated by the Sketch Engine indicated a greater consistency of patterning and sets of variables to the right collocates of 世界 *shi4jie4* than to its left. Since collocates at the L4 position of it were too sparse, they were not included in Table 1.

**Table 1:** Left collocates of 世界 *shi4jie4*.

**L1**				**L2**			**L3**		
**Rank**	**Collocate**		**LogDice**	**Collocate**		**LogDice**	**Collocate**		**LogDice**
1	全	All	8.799	成为	Become	8.456	这个	This	8.195
2	当今	Today	8.579	走向	Walk to	8.208	中国	China	7.685
3	走向	Walk to	8.127	这个	This	8.055	我国	Our country	6.824
4	成为	Become	8.105	乃至	And even	7.656	已	Has	6.792
5	这个	This	7.953	居	Rank	7.605	让	Let	6.703
6	居	Rank	7.589	来自	Come from	7.516	作为	As	6.566
7	来自	Come from	7.318	向	Toward	7.29	拥有	Own	6.526
8	乃至	Reach to	7.231	第二	Second	7.002	认识	To know	6.519
9	整个	Whole	7.191	面向	Turn toward	6.969	对	For	6.501
10	届	A classifier of period	7.12	第三	Third	6.926	改造	Transform	6.469
11	占	Take	7.021	在	In/at/on/around	6.861	改变	Change	6.46
12	内心	Mind	6.943	是	BE	6.81	西方	Western	6.366

At the L1 position, a semantic set, including the frequently occurring member 目前 *mu4qian2* (at present) (6.919) and the less-frequently occurring member (not distributed in the table) 当时 *dang1shi4* (at that time) (4.996), was grouped into a semantic association of TIME. At the L2 position, a series of ordinal words [e.g., 第二 *di4er4* (second) and 第三 *di4san1* (third) and verbs (e.g., 乃至 *nai3zhi4* (reach to) and 居 *ju1* (rank)] formed a meaning associated with RANK. Collocates at the L3 position to 世界 *shi4jie4* seemed to be associated with a semantic meaning of CHANGE [e.g., 改造 *gai3zao4* (transform) and 改变 *gai3bian4* (change)], and REGION [e.g., 中国 *zhong1guo2* (China), 我国 *wo3guo2* (our country), and 西方 *xi1fang1* (Western)].

At the L1 position, *world* and 世界 *shi4jie4* shared the collocates of *whole*/全 *quan2* (or 整个 *zheng3ge4*) (whole) and *today's*/当今 *dang1jin1* (today's). A slight difference was that *today's* had a lower collocational strength of co-occurring with *world* and thus is not distributed in Table 2; however, it also belonged to the semantic association of POSITIONING IN TIME of *world*.

**Table 2:** The left collocates of *world*.

**L1**			**L2**		**L3**	
**Rank**	**Collocate**	**LogDice**	**Collocate**	**LogDice**	**Collocate**	**LogDice**
1	Third	9.616	Around	9.616	Parts	7.853
2	Second	9.503	Throughout	9.503	Rest	7.793
3	First	8.832	Outside	8.832	All	6.905
4	Real	8.071	Over	8.071	Anywhere	6.708
5	Outside	8.032	In	8.032	Countries	6.339
6	Arab	8.025	Of	8.025	Best	6.304
7	Whole	7.311	Round	7.311	End	6.125
8	The	7.294	Across	7.294	Largest	6.033
9	Modern	7.099	Into	7.099	Modern	7.099
10	New	6.781	During	6.781	New	6.781
11	Third	6.677			Third	6.677
12	Western	6.67			Western	6.670
13	Natural	6.63			Natural	6.630
14	Developing	6.572			Developing	6.572
15	Second	6.548			Second	6.548

Next, we consider the second position to the left side of *world* and 世界 *shi4jie4*. Table 2 reveals that prepositions predominantly occupied the L2 position of *world*; this phenomenon also occurred at the L2 position of 世界 *shi4jie4*. Chinese is not a language with many variants of prepositions. T 在 *zai4* at L2 to 世界 *shi4jie4* could be treated as equivalent to different prepositions in English. Thus, we could conclude that 世界 *shi4jie4* had a strong colligational preference for occurring with prepositions at the L2 position, in the same way as did *world*.

In addition to the colligational behavior of co-occurring with prepositions, we discovered that 世界 *shi4jie4* was likely to occur with ordinal items, such as 第二 *di4er4* (second) and 第三 *di4san1* (third), at the L2 position. These lexical items occurred at the L2 position to 世界 *shi4jie4* rather than at the L1 position (as they did with *world)* simply because the classifier 次 *ci4* occurred between them. In Chinese, 第二次 *di4er4ci4* and 第二次 *di4san1ci4* are fixed co-occurrences that correspond to the word combinations *the second* and *the third*, respectively, in English.

Next, we looked at collocates on the right sides of the pair. Because the collocates to the right side of *world* are too sparse to generalize a semantic meaning, the comparison of right side collocates was only restricted to the two-word span. The collocates at R1 and R2 positions are distributed in Tables 3, 4.

**Table 3:** The right collocates of *world*.

**R1**			**R2**	
**Rank**	**Collocate**	**LogDice**	**Collocate**	**LogDice**
1	War	10.706	II	10.706
2	Cup	9.998	Title	9.998
3	Bank	8.648	Countries	8.648
4	Champion	7.560	Championship	7.56
5	War	7.478	Market	7.478
6	Largest	7.360	Championship	7.36
7	Trade	6.886	Economy	6.886
8	Countries	6.876	Championships	6.876
9	Health	6.766	Champions	6.766
10	Economy	6.702	Where	6.702
11	Broadcasts	6.683	Biggest	6.693
12	Title	6.651	Most	6.651
13	Market	6.693	Which	6.585
14	Champions	6.585	Championship	6.477
15	Championship	6.477		

**Table 4:** Right collocates of 世界 *shi4jie4*.

	**R1**			**R2**		
**Rank**		**Collocate**	**LogDice**		**Collocate**	**LogDice**
1	一流	First	8.195	一流	First	8.015
2	公认	Recognized	7.685	公认	Recognized	7.891
3	大战	War	6.824	最	Most	7.602
4	上	Up	6.792	著名	Famous	7.344
5	著	Famous	6.703	唯一	Only	7.3
6	闻名	Renowned	6.566	大战	War	7.255
7	和平	Peace	6.526	上	Up	7.164
8	顶级	Top	6.519	最早	The earliest	7.038
9	末日	End	6.501	遗产	Heritage	6.58
10	知名	Well-known	6.469	和平	Peace	6.506
11	领先	Leading	6.46	顶级	Top	6.449
12	500		6.366	闻名	Renowned	6.401

At the R1 position of *world*, the collocates with higher LogDice scores were *war, Cup, Bank, Champion, war, largest, trade, countries, Health, economy, Broadcast, title, market, champions*, and *championship*. These collocates showed a semantic association that might be labeled as COMPETITION, represented by the words *cup, Champion, champions*, and *championship*; ECONOMY, represented by *Bank, trade, economy*, and *market*; and RANK, dominantly represented by the superlative *largest* and supplemented by members with lower LogDice scores, such as *biggest* (6.324), *greatest* (5.776), and *oldest* (4.588). At the R2 position, three semantic associations could be grouped, including COMPETITION, represented by the words *Champion, Championship*, and *champions;* ECONOMY, represented by *market* and *economy*; and RANK, represented by the superlatives *biggest* and *most*.

We now turn to the right side collocates of 世界 *shi4jie4*. Unlike collocates occurring at the R1 position for *world*, the top R1 collocates of 世界 *shi4jie4* demonstrated more variants, which were predominantly made up of adjectives, such as 一流 *yi1liu2* (first), 公认 *gong1ren4* (universally acknowledged), 著名 *zhu4ming2* (famous), 闻名 *wen2ming2* (renowned), and so forth. These candidates were generalized into semantic associations, such as FAME, represented by words of 公认 *gong1ren3* (recognized by the public), 著名 *zhu4ming2* (famous), 闻名 *wen2ming2* (renowned), and 知名 *zhi1ming2* (well-known), and FIRST CLASS, represented by 一流 *yi1liu2* (first), 领先 *ling3xian1* (leading), 顶级 *ding3ji2* (the best, the top), and *500* (indicating the top 500 enterprises in the world).

The frequent collocates of 世界 *shi4jie4* at the R2 position were mostly shared by *world* at its R1 position. This might be attributable to the frequent occurrence of 上 *shang4* (a character normally used after a noun in Chinese to specify a limit or boundary) at the R1 position. Together with 世界 *shi4jie4*, 上 *shang4* forms a typical, frequently occurring phrase, 世界上 *shi4jie4shang4*, which corresponds to the frequently occurring English phrase, *in the world*, in most contexts. In addition to these shared collocational members, collocates appearing at the R2 position of 世界 *shi4jie4* were likely to be colligated (and collocated) with RANK, represented by 最 *zui4* (most) *and* 最早 *zui4zao3* (earliest).

Comparing collocates at different positions to the node pair indicated that a similar semantic meaning might be presented with different collocates in the two languages. The collocates could differentiate in terms of their positions (e.g., left or right) relative to the nodes and distance (e.g., number of tokens) from the nodes. For example, the two nodes share a semantic meaning of RANK. However, the collocates contributing to this meaning occurred respectively at the L1 position to *world* and the L2 position to 世界 *shi4jie*.

### The contrast of clusters in respect of their collocational and semantic associational behaviors

As shown in previous tables, the node pair *world* and 世界 *shi4jie4* co-occurred with certain other words to form a certain semantic meaning. The clusters or word sequences made up of the words become loaded with the contexts and co-texts in which they occur. This is defined as “nesting” by Hoey (2005, p. 8), “where the product of a priming becomes itself primed in ways that did not apply to the individual words making up the combination.” According to the definition, a cluster may sometimes have specific collocations not shared by its component(s). For example, *winter* collocates with *in*, thus producing a phrase of *in winter*. But this phrase has its own collocations, separate from *in* and *winter*. In this section, I will observe two frequently used clusters containing *world* and 世界 *shi4jie4*, respectively. Their collocations and semantic associations with both sides are also considered.

Sketching clusters of *world* and 世界 *shi4jie4* within a four-word span yielded several instances. Among the whole, I selected the first two clusters (data were filtered according to the parameter of running words per million) as the focus of the study, namely, *in the world* (5,925 hits with 52.74 running words per million), *the second world war* (1,544 hits with 13.74 running words per million); and 世界上 *shi4jie4shang4* (in the world) (323,588 hits with 19.5 running words per million), 世界各地 *shi4jie4ge4di4* (every corner of the world) (194,045 hits with 11.69 running words per million). As evidenced by the MC value^2^ (53%), the English *in the world* has a very strong tendency to correspond to 世界上 *shi4jie4shang* in Chinese. In the present study, we treat *in the world* and 世界上 *shi4jie4shang* as highly translatable to each other.

#### The collocations of *in the world*

Table 5 shows that all but two of the collocates of *in the world* formed a semantic association of SUPERLATIVE (and also a colligation) made up of words, such as *largest, biggest*, best, *greatest, finest, richest, oldest*, and *most*. The tendency of *in the world* to occur with SUPERLATIVE was far stronger than that of the single-node word *world*. As noted previously, SUPERLATIVE was also a kind of colligation. Although we will exert a section to discuss the colligational behavior of *world* and 世界 *shi4jie4*, at this stage, we will talk about the preferences/aversions of the two clusters at the nominal group level, while leaving other colligational characteristics aside.

**Table 5:** The collocates of *in the world* within a four-word span.

**Rank**	**Collocates**	**Frequency**	**LogDice score**
1	Anywhere	230	9.588
2	Largest	222	9.287
3	Cup	159	8.622
4	Biggest	122	8.581
5	Best	453	8.568
6	Greatest	102	8.269
7	Finest	67	8.171
8	Richest	52	8.05
9	Oldest	57	7.985
10	Most	672	7.876

Using the “getting a randomized sample” function on Sketch Engine, we obtained a sample of 1,000 instances containing *in the world*. The qualitative annotation showed that *in the world* has a strong preference (73%) for occurring as a postmodification in a nominal group.

#### The collocations of 世界上 *shi4jie4shang4 (in the world)*

Examining the collocates occurring immediately within the four-word span (see Table 6) to the node cluster, we found the typical meaning associated with it was also SUPERLATIVE, composed by collocates such as 最长 *zui4da4* (the longest), 最早 *zui4zao3* (the earliest), 最大 *zui4da4* (the biggest), 最小 *zui4xiao4* (the smallest), 最快 *zui4kuai4* (the quickest), 最好 *zui4hao3* (the best), etc. This semantic meaning was also echoed by its English corresponding equivalence *in the world*. Nevertheless, the qualitative data showed that, while *in the world* is likely to be used in the postmodifying position, 世界上 *shi4jie4shang4 (in the world)* has a positive tendency (69%) to occur at the premodifying position in a nominal group.

**Table 6:** The collocates of 世界上 *shi4jie4shang4 (in the world)* within a four-word span.

**Rank**		**Collocates**	**Frequency**	**LogDice score**
1	最先进	The most advanced	5,702	8.836
2	独一无二	The only one of its kind	3,756	8.080
3	最长	Longest	2,477	7.762
4	最早	Earliest	3,471	7.394
5	最大	Biggest	11,598	7.224
6	最小	Smallest	2,027	7.189
7	公认	Universally acknowledged	2,077	7.120
8	迄今	So far	1,966	6.954
9	最美	The most beautiful	2,072	6.856
10	第一	First	31,819	6.579
11	最快	Fastest	1,411	6.575
12	最	Extremest	53,474	6.569
13	活在	Living in	1,027	6.493
14	号称	Claimed to be	1,336	6.489
15	为止	To the end	2,137	6.470
16	最好	Best	5,561	6.454
17	伟大	Greatest	2,354	6.306
18	这个	This	34,202	6.270
19	为数不多	Few	850	6.253
20	当今	Contemporary	1,635	6.239

The commonality of associating *in the world*/世界上 *shi4jie4shang4* with the same meaning suggested that the two speech communities of English and Chinese conventionalized similar routes on the two phrases. Psychologically, speakers of English and Chinese recognize the polar meaning represented by the two clusters. People repeatedly encounter using the word sequence *in the world*/世界上 *shi4jie4shang4* as the highest level of their evaluation system. The combinations are repeatedly used in a discourse. A listener or reader may expect certain combinations when listening to or reading a sentence or text consisting of the combination. The frame they construct allows them to process the text more effectively or quickly.

#### The collocations of *the second world war*

Table 7 shows the collocates within four words to the left and right of the cluster, *the second world war*. These collocates are then grouped into three major semantic associations. The first is that of TIME, represented by collocates such as *During, after, 1939, 1940's, 1920's, 1945, 1931*, etc. The second semantic association is concerned with PARTICIPANTS of *the second world war*, represented by collocates of *Nazi*, Cossacks, *Germans, Hitler, fascist*, etc. The third semantic meaning is associated with the war's AFTERMATH, such as *aftermath, Depression*, repatriation, *devastation*, etc.

**Table 7:** The collocates of *the second world war* within a four-word span.

**Rank**	**Collocates**	**Frequency**	**LogDice score**
1	Outbreak	29	8.584
2	During	63	7.871
3	During	233	7.661
4	Aftermath	11	7.361
5	Since	208	7.338
6	Since	29	6.583
7	Longest	7	6.483
8	Requisitioned	4	6.357
9	After	239	6.353
10	Interned	4	6.336
11	1945	8	6.287
12	Bombing	6	6.266
13	Cossacks	4	6.263
14	Before	16	6.260
15	End	110	6.238
16	Nazi	5	6.173
17	After	46	6.171
18	Victorious	4	6.166
19	1930s	7	6.159
20	Wrought	4	6.134

Compared with *the second world war*, there were only 4.66 running words per million of 第二次世界大战 *di4er4ci4shi4jie4da4zhan4* (the second world war) in zhTenTen 11. Although this word sequence was not used as frequently and commonly as in English, its collocates within a four-word span show a strong resemblance with *the second world war*. Table 8 displays some of the members contributing to the meaning of TIME, including words such as *1945, 1939, the ending period*, etc.; and some of the members contributing to the meaning of PARTICIPANTS, including words such as *allies, fascist, German army*, etc.

**Table 8:** The collocates of 第二次世界大战*di4er4ci4shi4jie4da4zhan4* (the second world war) within a four-word span.

**Rank**	**Collocate**		**Frequency**	**LogDice score**
1	大战	War	37,186	11.240
2	第二	Second	37,186	7.771
3	纳粹	Nazi	324	6.944
4	1945年	The year of 1945	347	6.877
5	末期	Ending period	296	6.641
6	盟军	Ally	174	6.614
7	1939年	The year of 1939	187	6.445
8	战败	Be defeated	166	6.381
9	爆发	Outbreak	1,682	6.362
10	反法西斯	Anti-fascist	116	6.312
11	法西斯	Fascist	164	6.029
12	Victoire		74	6.022
13	停战	Armistice	92	5.883
14	次	Times	37,085	5.864
15	德军	German army	122	5.810
16	同盟国	Allied nations	71	5.769
17	回忆录	Memoirs	110	5.716
18	希特勒	Hitler	129	5.703
19	战犯	War criminals	85	5.699
20	战胜国	Victorious nations	63	5.661

#### The collocations of 世界各地 *shi4jie4ge4di4* (every corner of the world)

Now, we consider the most frequently used cluster containing 世界 *shi4jie4*. The collocates appearing to both sides of 世界各地 *shi4jie4ge4di4* (every corner of the world) are displayed in Table 9.

**Table 9:** The collocates of 世界各地*shi4jie4ge4di4* (every corner of the world) within a four-word span.

**Collocates**		**Frequency**	**LogDice score**
各地	Every corner	194,045	12.439
遍布	To spread all over	4,681	8.613
来自	From	40,058	8.591
遍及	Be found everywhere	1,780	7.749
销往	To export	1,510	7.605
远销	To sell something to a distance place	1,447	7.540
吸引	To attract	8,026	7.103
游客	Travelers	6,183	6.886
汇集	To compile	982	6.453
乃至	And even	1,703	6.363
爱好者	Hobbyist	1,351	6.300
足迹	Foot-mark	661	6.148
汇聚	To collect	988	6.138
美食	Tasty food	1,968	6.125
走遍	To travel around	493	6.080
分布	Be distributed	2,228	6.044
畅销	Best sold	758	5.999
成千上万	Thousands of	498	5.948
运往	Be transported to	415	5.903
搜罗	To collect	374	5.796

The data in the table suggest that the general semantic meanings associated with the word sequence are TRADE, represented by collocates of 销往*xiao1wang3* (to export*)*, 远销*yuan3xiao1* (to sell something to a distance place*)*, 畅销 *chang4xiao1* (best sold), etc.; and TRAVELING, represented by 游客 *you2ke4* (travelers*)*, 爱好者 *ai4hao4zhe3* [hobbyist (of traveling)], 走遍 *zou3bian4* (to travel around*)*, etc. The English counterpart corresponding to 世界各地 *shi4jie4ge4di4* has many alternatives. Among the many, we chose to observe *anywhere in the world* (196 hits with 1.74 per million words), which had the highest MC value (34%). The data showed that English speakers are very unlikely to prime this phrase. Apart from the low frequency, the collocates appearing to both sides of *anywhere in the world* were too dispersed to be grouped into any kind of semantic association.

Hoey illustrated in LPT that we hold in our minds elaborate networks of possible co-occurrence patterns that are primed for particular contexts or situations. Per the theory, “mental concordance” stored in our mind sparks the expectancies we refer to build up discourses. Through repeated encounters with a word combination or word sequence in a particular textual and social context, we begin to identify it as collocation. Repeated encounters with the collocation are then primed when we are exposed to similar contexts. The knowledge of a word's repeated encounters then contributes to people's sentential comprehension.

The present study's results coincide with psychological experiments concerning reading tasks (e.g., Reali and Christiansen, 2007), in that the frequency of being exposed to words or sequences of words repeatedly used in similar contexts could influence readers' expectations. This observation provides two psychological insights for cross-linguistic studies, the first being the insight into the definition of corresponding equivalence. The cross-linguistic equivalence between English and Chinese should reside in a unit of meaning rather than single-word-to-word translation. As the above data show, restricting the correspondence in one or two lexical items may not reflect the corresponding meaning across the two languages. It seems all the more evident that the corresponding meaning is inseparably linked up with a phraseological patterning of the node item. These patterns or clusters are the habitual and typical expressions of a language. From these frequently used clusters, we can infer a speech community's habitual ways of conceptualization, which may determine their ways of sentential comprehension.

The second insight concerns second language acquisition. The difficulty of learning a target language is believed to depend preliminarily on the context in which the target language pattern is similar to or different from that of a learner's first language. This finding is in line with Jantunen and Brunni (2013), who showed that a learner's mother tongue background does not seem to affect the ability to produce correct word forms. Corresponding equivalence resides in a more complex pattern than simple word-to-word translation. Teachers can crack in harmonious primings directly or recommend that students read more authentic materials to find the conflicts between the learners' primings and the common primings shared by the community in which their listeners or readers live.

Hoey (2005, p. 8) noted that priming can be the fullest form of describing Giddens (1979) discussion of the relationship between human agency and social structure, where each action reproduces the structure, and the structure shapes the individual action. Per Hoey, “priming leads to a speaker unintentionally reproducing some aspect of the language, and that aspect thereby reproduced, in turn, primes the hearer.” In Hoey's theory and other research taking LPT as a theoretical framework, the context that may influence an individual's priming is restricted to written or spoken discourse; however, since LPT intends to describe corpus data from a psychological dimension, we could not neglect the context of society taking upon an individual's priming.

Due to the identical psychological representation of the physical world, both English and Chinese speakers apply *in the world*/世界上 *shi4jie4shang4* to express the highest level in their evaluation hierarchy, as indicated from the quantitative results obtained earlier. In addition, the semantic priming represented by the collocates surrounding the node clusters within a four-word span is identical. Apart from the congruence, some divergence was also found. This divergence, in some cases, is strictly opposed to each other. While BNC shows English speakers' preference for applying *the second world war*, zhTenTen11 indicates Chinese speakers' strong aversion to picking up this cluster. While zhTenTen11 shows a preference for deploying 世界各地 *shi4jie4ge4di4* (every corner of the world), BNC demonstrates no significant tendency to use its counterpart.

A language user within a speech community may acquire the primings of a word sequence first or the primings of the individual word first. There is no difference in principle between the two ways of first language acquisition. However, it is assumed based on data obtained above that the language production and the process of comprehending a phrase or sentence could be influenced by the knowledge of collocation on that word. If the “nonce” primings encountered by the speaker of a language are compatible with the primings stored in their brain, the matching semantic associations are then primed (the procedure can be *vice versa*). In this sense, the comprehension speed of the language can be accelerated.

### The contrast of colligational behaviors between *world* and 世界 *shi4jie4*

Colligation in LPT characterizes itself by claiming that every word prefers or avoids appearing at a certain position, grammatical function, or textual position. The present study compares the syntactic behaviors of *world* with those of other, apparently similar nouns discussed in Hoey's (2005, p. 46–49), namely, *consequence* (1,615 hits), *question* (300 hits), *preference* (300 hits), *aversion* (204 hits), and *use* (300 hits). For 世界 *shi4jie4*, I selected five lexical items from the first paragraphs of Text 01 in the LCMC, namely, 结果 *jie2guo3* (result, 242), 问题*wen4ti2* (question, 451), 语言 *yu3yan2* (language, 355), 工业 *gong1ye4* (industry, 261), and 范围 *fan4wei2* (range, 141). They operated at a comparable degree of abstractness.

#### The general colligational profile of *world*

According to Hoey (2005, p. 44), “A noun will always be part of some group or other word sequence, and that group or word sequence will normally perform some function in a clause.” It is thus reasonable to examine the distribution of a noun in terms of its occurrences within a clause or group. This section observes the colligational behavior of *world* at the clausal level, and then at the group level. Four major clausal-level grammatical functions were considered in connection with *world*: as part of a Subject, as part of an Object, as part of a Complement, and as part of a prepositional phrase functioning as an Adjunct. Our use of these grammatical terms is generally in line with normal usage and the definitions in the *Collins Grammar Dictionary*, wherein Subject refers to “the noun group that refers to the person or thing that is doing the action expressed by the verb” (as Example 1 shows) and Object is defined as “a noun, noun phrase or pronoun that refers to a person or thing that is affected by the action of the verb (called the DIRECT OBJECT), or that the action is done to or for (called the INDIRECT OBJECT)” (as Example 2 shows). Complement, on the other hand, is defined as “an adjective group or noun group, which comes after the verb and describes or identifies the subject.” It normally follows the BE verb or other equative verbs, such as FEEL, BECOME, and SEEM (as Example 3 shows). Adjunct in the present study referred to the prepositional phrase, which is used to modify the clause. It could appear at either the beginning or the end of a sentence (as Example 4 shows). Instances that do not fit one of these four basic grammatical categories are simply analyzed as Other.

1) For Einstein, the physical **world [Subject]** was an incarnation of reason, which, although manifested in various laws and principles, was inaccessible to the human mind in its most profound depths.
2) Almost all the growth will occur in the cities of the developing **world [Object]**.
3) Now, the open systems is a very new **world [Complement]** to us.
4) CGE is the largest water group **in the world [Adjunct]**.

Table 10 reveals a clear negative colligation between *world* and the grammatical function of Adjunct. Only 14% of instances belonged to this function, almost three times less often than for *consequence*.

**Table 10:** A comparison of the grammatical distributions between *world* in a clause and that of five words discussed in Hoey (2005).

	**Part of subject**	**Part of object**	**Part of complement**	**Part of adjunct**	**Others**
*World*	27% (99/365)	33% (120/365)	19% (69/365)	14% (51/365)	7% (26/365)
*Consequence*	24% (383/1615)	4% (62/1615)	24% (396/1615)	43% (701/1615)	5% (74/1615)
*Question*	26% (79/300)	27% (26/300)	20% (60/300)	22% (66/300)	4% (13/300)
*Preference*	21% (63/300)	38% (113/300)	7% (21/300)	30% (90/300)	4% (13/300)
*Aversion*	23% (47/203)	38% (77/203)	8% (16/203)	22% (45/203)	8% (17/203)
*Use*	22% (67/300)	34% (103/300)	6% (17/300)	36% (107/300)	2% (6/300)

Regarding Complement, while the tendency of *world* to occur in this function was not as strong as that exhibited by *consequence*, it still occurred much more frequently than other parametric words, except for *question*.

Then, we continue to examine the colligational features of *world* at the nominal group level, which include three grammatical possibilities: occurring as the head of the nominal group (as example 5 shows), as a premodifier (as example 6 shows), or as part of the postmodification (as example 7 shows). For example:

5) They argue that the **world** has obligations under the Genocide Convention of 1948. **[world as head]**
6) The lad from a farm close to a one-horse stop called Peachester, near Brisbane, became a **world** figure with some of the best golf ever seen in the last round of a major championship. **[world as premodifier]**
7) Unfortunately, he cannot pick Ian Baker-Finch, one of the best known golfers in the **world**. **[world as part of the postmodification]**

I analyzed all nominal groups within the 365 instances containing the node word *world* from the FLOB. As before, its syntactic behavior was compared with that of the five words analyzed by Hoey (2005).

As Table 11 shows, *world* and *consequence* clearly differed in grammatical distribution, despite sharing the same colligational preference for the Subject function. *World* differed strikingly from *consequence* and the other four nouns in two ways. First, *consequence* strongly tended (98%) to occur as the head of its nominal groups, as did the other words (75–92%), whereas *world*'s tendency was only 33%. *World* occurred least frequently as part of the premodification among the three possibilities; it was overwhelmingly more likely to do so than the other five words, especially *consequence*, which showed almost no such tendency. Second, while *preference, aversion*, and *use* occurred in postmodification between two and 12 times more frequently than *consequence, world* showed an even stronger tendency, occurring 21 times more often. Within the 154 instances of *world* occurring as part of postmodification, 70% (108/154) occurred in the form of a prepositional phrase, dominantly represented by the prepositional phrase *in the world*, accounting for 34% of prepositional phrases (37 out of 108) and *on the world* (stage), accounting for 28% (30 out of 108).

**Table 11:** A comparison of the grammatical distribution of *world* in the nominal group with words studied by Hoey (2005).

	**Head of nominal group**	**Premodifier of nomical group**	**Part of the postmodification of the nominal group**
*World*	33% (119/365)	25% (92/365)	42% (154/365)
*Cosequence*	98% (1588/1615)	0.06% (1/1615)	2% (26/1615)
*Question*	92% (275/300)		8% (25/300)
*Preference*	84% (253/300)	3% (8/300)	13% (39/300)
*Aversion*	82% (167/203)	6% (12/203)	12% (24/203)
*Use*	75% (226/300)	1% (2/300)	24% (72/300)

The typical colligational behaviors of *world* at the nominal group level included:

1. a strong preference for being used as part of the postmodification of a nominal group;
2. a positive preference for appearing as part of the postmodification of a nominal group and proportionally frequently in the form, *in the world*;
3. a strong preference for being used as the premodification of a nominal group;
4. a strong aversion to being used as the head of a nominal group.

#### The general colligational profile of 世界 *shi4jie4*

Five hundred thirty-five instances of 世界 *shi4jie4* were analyzed to see whether they occurred as part of the Subject, as part of the Object, as part of the Complement, or as part of a prepositional phrase functioning as an Adjunct, using the same methodology used to analyze the word *world*.

Our use of the grammatical categories for Subject (Example 8), Object (Example 9), Complement (Example 10), and Adjunct (Example 11) was in line with the normal use defined in *Grammar and Rhetoric of Speech Act*.

8)   当今   世界各国   **[Subject]**   对
  dang1jin1   shi4jie4ge4guo2   dui4
  Today   every nation of the world   toward
  节能降耗   达成了   共识。
  jie2neng2jiang4hao4   da2cheng2le   gong4shi2
  energysaving   reach   common sense

Today, all the nations in the world have reached a common sense toward the issue of energy saving.

9)   其实   是   最伟大的人,   他们
  qi2shi2   shi4   zui4wei3da4deren2   ta1men
  Actually   is   the greatest people   they
  改变   世界 **[Object]**。
  gai3bian4   shi4jie4
  change   the world

Actually, it is the greatest people who have changed the world.

10)   皮肤病   是   世界 **[Complement]**公认的
  pi2fu1bing4   shi4   shi4jie4gong1ren4de
  Skin diseases   are   world recognized
  常见疾病   及   多发病。
  chang2jian4ji2bing4   ji2   duo1fa1bing4
  Normal diseases   and   frequently occurring diseases

It is universally acknowledged that skin diseases are kinds of normal and frequently-occurring diseases.

11)   在世界范围内   我们   要
  zai4shi4jie4fan4wei2nei4   wo3men   yao4
  In the range of the world   we   need
  遵守   国际秩序   维护
  zun1shou3   guo2ji4zhi4xu4   wei2hu4
  abide by   international order   maintain
  国家   形象。
  guo2jia1   xing2xiang4
  national   image

In the range of the world, we must abide by the international law and maintain our national image.

As distributed in Table 12, 世界 *shi4jie4* was quite strikingly different from the other five nouns in terms of grammatical functions. We found positive and negative colligations with the clausal functions with which 世界 *shi4jie4* was likely to be associated; these preferences and aversions are summarized as follows:

1. There was a clear positive colligation between 世界 *shi4jie4* and the grammatical function of Object, accounting for over half the total instances extracted from the LCMC.
2. There was also a positive colligation between 世界 *shi4jie4* and the grammatical function of Adjunct. Although the percentage was not strikingly higher than for several of the other nouns [excluding 范围 *fan4wei2* (range)], 世界 *shi4jie4* displayed a relatively stronger tendency to be associated with this function.

**Table 12:** A comparison of the grammatical distribution of 世界 *shi4jie4* in the clause with that of other nouns in the LCMC.

	**Part of subject**	**Part of object**	**Part of complement**	**Part of adjunct**	**Other**
*世界 shi4jie4 world*	14% (75)	58% (310)	7% (37)	20% (107)	1% (5)
*结果 jie2guo3 result*	36% (88)	23% (55)	9% (22)	18% (44)	14% (33)
*问题 wen4ti2 question*	22% (100)	56% (251)	11% (50)	6% (25)	6% (25)
*语言 yu3yan2 language*	24% (58)	19% (46)	43% (104)	10% (23)	4% (11)
*工业 gong1ye4 industry*	55% (144)	34% (90)	0	7% (18)	3% (9)
*范围 fan4wei2 range*	17% (23)	40% (56)	0	44% (62)	0

We now look at the colligational behavior of 世界 *shi4jie4* at the rank of the group or phrase. As with *world*, there are, in principle three grammatical possibilities for 世界 *shi4jie4* occurring within a nominal group: as the head of the nominal group in which it appears (as Example 12 shows), as part of the premodifier (as Example 13 shows), or as part of the postmodification (as Example 14 shows):

12)   当今世界   属于   激烈的
  dang1jin1shi4jie4   shu3yu2   ji1lie4de
  Today's world   belongs to   fierce
  市场竞争   当中。   
  shi4chang3jing4zheng1   dang1zhong1
  market competition   in
  **[**世界***   shi4jie4* occurs as a noun head]**

The world today is involved in fierce market competition.

13)   自然神   成为   当时
  zi4ran2shen2   cheng2wei2   dang1shi2
  The god of nature   become that   time
  主宰人们   内心世界的   主要
  zhu3zai3ren2men   nei3xin1shi4jie4de   zhu3yao4
  dominate people   inner world   major
  精神力量。
  jing1shen2li4liang4
  spiritual power
  **[**世界*** shi4jie4* occurs as part of premodifier]**

The God of nature had become the main spirit power for people's inner world at that time.

14)   上海光源   以世界   同类装置
  Shang4hai3guang1yuan2   yi3shi4jie4   tong2lei4zhuang1zhi7
  Shanghai light source   world   instruments alike
  最少的   投资   和   最快的
  zui4shao3de   tou2zi1   he2   zui4kuai4de
  least   investment   and   fast
  建设速度,   实现了   优异的   性能…
  jian4she4su4du4   shi2xian4le   you1yi4de   xing4neng2
  construction speed   realize   brilliant   performance
  **[**世界*** shi4jie4* occurs as postmodification]**

[The company of] Shanghai Light Resource had gained brilliant performance on similar devices in the world with the least investment and fast construction.

Table 13 shows that 世界 *shi4jie4* clearly differed from the other nouns studied in terms of its distribution in a nominal group. Those differences were much more significant and striking than other grammatical functions. First, while the other nouns most occurred frequently as heads in nominal groups, particularly 结果*jie2guo3* (result), 世界 *shi4jie4* occurred as the head much less often, indicating it tends to be used to specify noun heads and avoids being the center of attention. Second, 世界 *shi4jie4* had a very strong tendency to occur in the premodification function of a noun group, closely followed by 工业*gon1ye4* (industry). Third, none of the nouns under study showed a preference for occurring as part of a postmodification, except for 世界 *shi4jie4* and 问题 *wen4ti2* (question), which showed some small tendency to occur in this grammatical position. In summary, 世界 *shi4jie4* had a negative colligation for occurring as the noun head, and世界 *shi4jie4* a positive colligation for occurring as part of premodification.

**Table 13:** A comparison of the grammatical distribution of 世界 *shi4jie4* in the nominal group with that of the other five nouns in the LCMC.

	**Noun head**	**(As part of) premodification**	**Postmodification**
*世界 shi4jie4 world*	33% (177/535)	65% (348/535)	2% (11/535)
*结果 jie2guo3 result*	100% (242/242)	0	0
*问题 wen4ti2 question*	75% (338/451)	19% (85/451)	6% (28/451)
*语言 yu3yan2 language*	55% (195/355)	45% (160/355)	0
*工业 gong1ye4 industry*	46% (120/261)	54% (141/261)	0
*范围 fan4wei2 range*	92% (129/141)	8% (12/141)	0

Examination of instances containing *world* and 世界 *shi4jie4* reveals complex issues concerning its grammatical position in each function. Regarding grammatical functions, *world* and 世界 *shi4jie4* were positively primed as part of Object. Their divergence lies in the position they served in a nominal group. The word *world* was primed in favor of postmodification, while the word 世界 *shi4jie4* was primed in favor of premodification. At the nominal group level, the two nodes share a strong aversion to occurring at the noun head position.

The behavior of 世界 *shi4jie4* (and also *world*) was biased toward particular grammatical positions and against others when serving a particular grammatical function. The evidence also suggests that the preferences/avoidances are not always characteristics of the node's syntagmatic behavior and may interact with other such factors as collocation and semantic association. The complexity of the patterns identified in the study points to an interaction between units at different levels (e.g., collocation, colligation, and semantic association). Colligation data in the present study indicated that a word or word sequence is primed to occur in (or avoid) certain grammatical environments, particularly the grammatical function it serves and the position in a nominal group in this paper. The colligation priming is irrespective of its priming for collocation or semantic association. This finding collaborated with the “holistic storage” model postulated by Wray (2002), which refers to the combination of the way a word sequence is stored and retrieved as a whole and the way a word sequence is stored and retrieved with grammatical rules.

## Discussion

The essence of LPT says that a person's repeated exposure to contextualized instances of highly similar sequences results in his or her being primed to associate those sequences with the recurrent features of those contexts. Every word is primed due to the cumulative effects of an individual's encounters with that word. The accumulated encounters of a word in a certain context or co-text then formulate a speaker's knowledge of this word. Hoey applied the psychological concept “priming” to account for the recurrent co-occurrence of words. The psychological explanations of collocation and other linguistic features (e.g., grammars and semantics) account for the naturalness of language. Hoey said a psychological explanation of collocation and other features implies that “there is no rational basis for believing that everybody's primings are identical” (Hoey, 2017, p. x). Previous research on LPT seeks to apply and advance the theory by testing English or other Indo-European languages. As a genetically and typologically different language from English, Chinese provides plenty of testing grounds for validating the theory. However, little research was conducted in applying the theory in cross-linguistic studies. To fill this gap, the present study attempted to extend the theory from the dimension of cross-linguistic contrast, which was not found in the literature. It sought to apply LPT as an explanation for cross-linguistic similarities and differences. LPT answers why collocation came into being and approaches the acquisition, understanding, and production of language; however, it does not address why similar or different primings occur in two distinct languages. The causal explanation underlying cross-linguistic behaviors needs to be provided. To answer these questions, we used cultural psychology as a backbone and claimed that words were primed culturally. Data displayed in the previous section will be discussed and accounted for.

We have examined the differences and similarities between *world* and 世界 *shi4jie4* in terms of the three fundamental concepts defined in LPT. In general, their divergence lies in their congruence. Based on the data, we found that both *world* and 世界 *shi4jie4* prefer to occur in the grammatical function of Object. In both English and Chinese, Object refers to the person or thing affected by the verb's action. It normally serves as a sentence's Rheme. The finding indicated that English and Chinese speakers commonly recognize *world* as an entity on which action can be taken. However, a different picture can be drawn if we glance at the verb collocates occurring before world and 世界 *shi4jie4* when functioning as part of Object. While the verbs preceding *world* are *develop, change, dominate, explore*, etc., the verbs co-occurring before 世界 *shi4jie4* are 走向*zou3xiang4* (to walk to), 享誉 *xiang3yu4* (to be well-known by), 闻名 *wen2ming2* (to be renowned by), 成为 *cheng2wei2* (to become), 居 *ju1* (to rank), etc.

A large body of cross-cultural research has shown that Westerners generally meet daily challenges analytically, whereas traditional Chinese tend to take a holistic approach. The former approach refers to the “tendency to extract the underlying properties of an object or phenomenons from its context,” whereas the latter thinking style refers to the belief that “things are interconnected with each other, be this directly or indirectly” (Li et al., 2010, p. 156). The difference between the two ways of thinking resides in how they view human beings' connections with their contexts. The BNC language users may conceptualize the world based on the context and treat it as an entity that could be developed, changed, explored or even created. In contrast, the Chinese language users of zhTenTen 11 tend to adopt a *zhong yong* (the doctrine of the mean) relation with the world, ranking it at the top position of their evaluation system and wanting to “成为 *cheng2wei2* (to become)” part of it, “走向 zou3xiang4 (to walk to)” it, and be well-known by people in other parts of it to keep connecting with it.

Sections The contrast of overall collocational and semantic associational behavior between *world* and 世界 *shi4jie4*, The contrast of clusters in respect of their collocational and semantic associational behaviors, and The contrast of colligational behaviors between *world* and 世界 *shi4jie4* demonstrated the collocational and colligational behaviors of frequently used word sequences across the two languages. The data showed that both *world* and 世界 *shi4jie4* preferred to appear in the phrase of *in the world*/世界上 *shi4jie4shang4*. The collocates co-occurred within a four-word span of the two node phrases generalized a similar semantic meaning, i.e., SUPERLATIVE. However, the divergence resides in their preference for grammatical positions. While *in the world* was strongly primed to occur at the postmodification position, 世界上 *shi4jie4shang4* was primed to occur at the premodification position. We tentatively apply the cultural psychology concept of personal identity to explain this phenomenon. Personality, according to Fellmann (2017, p. 1,588), “...is often experienced by individuals as a kind of mental fluctuations.” It is latent in the individual and integrated society. Political and economic structures are important factors in society; however, the more important or core factor is “ideological frameworks” (Fellmann, 2017). Western people's view of personhood emphasizes separating oneself from others, while Asian people emphasizes human beings' fundamental connectedness to each other. The world of Confucius, like China, is “a patriarchal construction of cultural psychology in which hierarchy and geometrical order dominate” (Fellmann, 2017, p. 1,588). This way of thinking habitually positions the most senior, knowledgeable, or authoritative person or thing as the arbiter of truth or moral order. This “authority-minded” (Shi and Feng, 2010, p. 560) way of thinking influences China's ethical system of social hierarchy.

This cultural psychological pattern accounts for Chinese speakers' preference for placing 世界上 *shi4jie4shang4* at the position of premodifier to emphasize the following noun head, because 世界 *shi4jie4* was viewed as the most authoritative and powerful entity in the physical evaluation system. An example is the Chinese way of writing an address, which places “China” at the very beginning, followed by subordinate levels of governance. In contrast, Western countries are more likely to be open societies, in direct opposition to the dominant culture of closed or traditional societies. They view themselves as independent of collectives, are less likely to recognize authorities, and prioritize their preferences, needs, and goals. This cultural psychological pattern could account for their habitual application of *in the world* at the post-position to the noun head, because they do not recognize *world* as an authority to be privileged over the noun head.

The data collection sections found no unique word sequences in the two languages. The shared clusters differ in terms of either frequencies or collocates appearing adjacent to them. For example, as shown in Section The contrast of clusters in respect of their collocational and semantic associational behaviors, the *second world war* pair was found in both languages. However, co-occurrence counts and LogDice showed a cross-linguistic variation in users' frequency and likelihood of picking up this sequence. English speakers strongly tend to use the word sequence in a semantic meaning of war PARTICIPANTS. Although occasionally associating this sequence with a similar meaning, Chinese speakers prefer applying it in the context of talking about the war's AFTERMATH and tend to avoid using it in their daily communication. While English language users demonstrate a strong preference for picking up the word sequence of *the second world war*, Chinese speakers are strongly primed to use the word sequence of 世界各地 *shi4jie4ge4di4* (every corner of the world) and prefer to associate it with a meaning of TRAVELING. Its English equivalent could be found in BNC, but with rather low frequency. This phenomenon could be accounted for from the perspective of individualism-collectivism, one of the most heavily researched dimensions of cultural psychology. The essence of individualism is the belief that the self is a self-contained, independent entity (Markus and Kitayama, 1991 cited in Chiu and Hong, 2005, p. 26); the essence of collectivism is the belief that the self is interdependent with some ingroup. Yuki (2003) further distinguished between the collective self and the relational self, with the former referring to a depersonalized self-defined in terms of prototypical properties shared among group members, and the latter defined by enduring connections and role relations with significant others. He then argued that the relational self is more characteristic of East-Asian collectivism. As the region's largest country, China undoubtedly characterizes itself with this feature to the largest degree. Chinese people emphasize ingroup connectedness or *Guanxi*. Chang and Lee (2012, p. 298) noted that “The Chinese live in a world with finely defined layers of relationships that make in- and out-groups differentiate gradated rather than binary.” To fix a perfect personal identity, the Chinese must maintain harmonious relations with others, including physical environment or psychological context. Therefore, they TRAVEL the world to obtain more understanding about the living environment to build better relations. In comparison, the English speakers studied seemed driven by their personal goals, emphasizing rational analysis in their relationship with the outside world or others, rather than intuition.

Two key factors of LPT are encountering and accumulating. The cumulative effects of an individual's encounters decide the possibilities of later priming. Each person in a speech community has a unique language that harmonizes with the other speakers to a considerable degree, constructing a language unique to that speech community. However, LPT fails to account for the reasons underlying speakers' decisions in the harmonizing process. Psychologically, a sense of belonging is the fundamental motive behind this behavior. One's participation in a culture is a means of fulfilling one's need to belong. Chiu and Hong (2005, p. 130) noted that a defining feature of a culture group is “the knowledge tradition believed to be mutually shared by the members of the cultural group.” When the need to belong to a group is activated, people who identify strongly with the ingroup will be motivated to adhere to its shared cultural tradition. People tend to view themselves as group members rather than separate individuals. Their need to belong to the ingroup is strengthened either by their realizing that they are different from others or not different from members of the ingroup (Chiu and Hong, 2005). This sense of belongingness explains the priming process in that individuals try to append their unique language usage to their culture's core values, thus formulating that speech community's unified priming.

## Conclusion

The study explored an effective way to apply LPT to explain cross-linguistic similarities and differences between English and Chinese. LPT is proposed based on the belief of “psychological association” between words and the mind. By making an analogy between mental concordance and computer concordance, Hoey applied corpus data to indicate the kinds of priming features present for language users. Gries (2012, p. 51) noted that data obtained from corpus linguistics “...are much concerned with things having immediate psycholinguistic and/or cognitive-linguistic relevance.” The data showcased in this paper align with much previous psycholinguistic research. The high frequency of using the clusters or word sequences across the two languages coincides with the “frequency effects” in psycholinguistics. Psycholinguists acknowledge a link between frequently used lexical items and features that trigger activation. By repeatedly encountering a word in a particular context, an individual's knowledge of the word is accumulated; “As social beings and as an integral part of all our animate and inanimate surroundings, we are touched, influenced, and formed by what we are exposed to” (Pace-Sigge, 2013, p. 27). The quantitative data in the present study show that English and Chinese speakers' cumulative effects are influenced by the regularity with which they are exposed to the word and their interactions with their societies. Chinese and English share similarities in collocation, semantic meaning, and colligation, but different priming characteristics, as demonstrated by their tendencies.

First, the quantitative data on collocations and clusters indicate that speakers in both languages have preferences for or repulsions against picking up a particular node and word sequence. They may be primed to associate similar meanings to the node but retrieve different words from their brain. Second, speakers in both languages make distinctive local syntactic options. The colligational data showcased syntactic option tendencies made regarding the node word, in line with lexicalist parsing theories in psycholinguistics, which holds that the recognition of a word or word sequence involves activating its grammatical option to “guide a further combinatory process” (Novick et al., 2003, p. 58). Speakers in distinct speech communities may have similar choices for grammatical functions but differ in their positional appearance choices. Language users' “mental concordances” are created through priming, and one could assume that continued encounters reinforce lexical and syntactical attachments to a particular lexical item in our neural network.

Based on the empirical data drawn from the corpora, we propose that words, in addition to being collocational, semantic associational, and colligational primed, are also culturally primed. To our knowledge, no research has been undertaken to apply or advance LPT in a cross-linguistic context between two genetically different languages, such as English and Chinese. Within the scope of cross-linguistic studies, there is no research accounting for the reasons behind cross-linguistic similarities and differences. The study attempted to apply LPT to interpret the lexical and grammatical features demonstrated by the two languages from a psychological perspective. Additionally, it extends the theory by considering the concept of cultural psychology. As Kashima (2015, p. 11) said, “Culture is neither a savior nor a nemesis; culture is a tool...it helps us to do what it is meant to do.” Corpus linguists and psychologists are urged to develop a better understanding of a person and his or her culture.

## Data availability statement

The original contributions presented in the study are included in the article/supplementary material, further inquiries can be directed to the corresponding author/s.

## Author contributions

The author confirms being the sole contributor of this work and has approved it for publication.

## Conflict of interest

The author declares that the research was conducted in the absence of any commercial or financial relationships that could be construed as a potential conflict of interest.

## Publisher's note

All claims expressed in this article are solely those of the authors and do not necessarily represent those of their affiliated organizations, or those of the publisher, the editors and the reviewers. Any product that may be evaluated in this article, or claim that may be made by its manufacturer, is not guaranteed or endorsed by the publisher.

## Footnotes

^1^When applying terms like “preference”/“avoidance,” we refer to the psychological preferences of the people using the word. Referring to preferences/avoidances in this way is a more economical way of describing the lexical behaviors found in English and Chinese. The primings indicated through the corpus data are not intended to suggest that words have preferences/avoidances themselves; rather, it is a simplified way of expressing the idea-demonstrated tendencies of the word (or word sequence) users.

^2^Mutual Correspondence value. This value was firstly introduced by Altenberg (1999). MC values of clusters containing the nodes were calculated based on the author's series of self-built datasets. If a corresponding pair has an MC value of more than 30%, it would be treated as strong equivalents in cross-linguistic or translational studies.
